# A Review of Hematological Complications and Treatment in COVID-19

**DOI:** 10.3390/hematolrep15040059

**Published:** 2023-10-13

**Authors:** Armand N. Yazdani, Arian Abdi, Prathosh Velpuri, Parth Patel, Nathaniel DeMarco, Devendra K. Agrawal, Vikrant Rai

**Affiliations:** 1 College of Osteopathic Medicine of the Pacific, Western University of Health Sciences, Pomona, CA 91766, USA; 2 Department of Translational Research, Western University of Health Sciences, Pomona, CA 91766, USA

**Keywords:** COVID-19, COVID therapy, hematological events, COVID-19 management

## Abstract

COVID-19, caused by SARS-CoV-2, and its variants have spread rapidly across the globe in the past few years, resulting in millions of deaths worldwide. Hematological diseases and complications associated with COVID-19 severely impact the mortality and morbidity rates of patients; therefore, there is a need for oversight on what pharmaceutical therapies are prescribed to hematologically at-risk patients. Thrombocytopenia, hemoglobinemia, leukopenia, and leukocytosis are all seen at increased rates in patients infected with COVID-19 and become more prominent in patients with severe COVID-19. Further, COVID-19 therapeutics may be associated with hematological complications, and this became more important in immunocompromised patients with hematological conditions as they are at higher risk of hematological complications after treatment. Thus, it is important to understand and treat COVID-19 patients with underlying hematological conditions with caution. Hematological changes during COVID-19 infection and treatment are important because they may serve as biomarkers as well as to evaluate the treatment response, which will help in changing treatment strategies. In this literature review, we discuss the hematological complications associated with COVID-19, the mechanisms, treatment groups, and adverse effects of commonly used COVID-19 therapies, followed by the hematological adverse events that could arise due to therapeutic agents used in COVID-19.

## 1. Introduction

An increasing prevalence of SARS-CoV-2 (COVID-19), including its variants, in the last four years has resulted in more than 64 million cases and 1.5 million deaths. This has led to an increased financial and socioeconomic burden [1]. COVID-19 is characterized symptomatically by cough, fever, anosmia, ageusia, and cardiovascular, neurological, psychiatric, gastrointestinal, and hematological manifestations. Common hematological abnormalities include thrombocytopenia, lymphopenia, altered coagulation, and disseminated intravascular coagulation (DIC) [1,2,3,4]. Following hematological abnormalities during COVID-19 infectivity longitudinally during the disease course is critical because it not only allows access to the treatment response but also the disease severity. For instance, patients with severe COVID-19 experience increased prothrombin time and D-Dimer, which are both associated with increased disease severity and mortality [1]. As new variants are constantly emerging and new treatment modalities are being put on the market, there is a rising concern about how COVID-19 treatment plans can cause detrimental hematological side effects. Patients with hematological malignancies experience more severe COVID-19 symptoms and higher morbidity and mortality [5]. In this review, we discuss the current medical treatment guidelines for COVID-19 and dissect the hematological response patients may experience to some of the widely used pharmaceuticals.

## 2. Material and Methods

A literature search was conducted using PubMed and Google Scholar to identify the background, mechanism, treatment usage, and side effects of pharmaceutical agents commonly used in the past to treat COVID-19. The keywords COVID-19, SARS-CoV-2, clinical findings, signs and symptoms, hematological effects, and drug interactions were used to search the literature. The selections were based on the article title and abstract, following which the full-text article was critically reviewed and the information included in this article. The findings from case reports, case series, and systemic reviews are summarized and discussed. The article search was focused on the published articles suitable for this review after the onset of COVID-19. 93 articles published in the last 4 years and 5 articles published before 2020 were included in the review. The duplicate, abstract-only, and non-English articles were removed during the literature search following PRISMA guidelines.

## 3. Hematological Complications 

### 3.1. Thrombocytopenia

**Figure 1:**
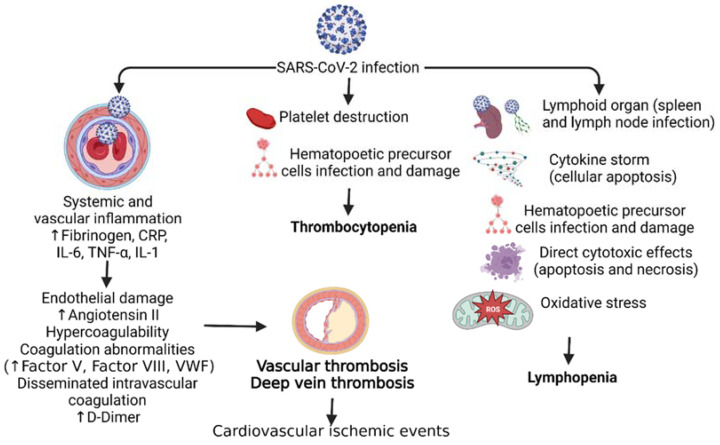
Pathophysiology of hematological disorders in SARS-CoV-2 (COVID-19) infection. SARS-CoV-2 infection leads to increased systemic vascular inflammation, platelet destruction, and infection of the lymphoid organs resulting in cardiovascular ischemic events, thrombocytopenia, and lymphopenia respectively. C-Reactive protein (CRP), interleukin (IL), tumor necrosis factor (TNF)-α, and Von Willebrand factor (VWF).

Thrombocytopenia occurring in 5–21% of COVID-19 patients is more severe in patients with more severe disease [1]. There have been cases of pseudo-thrombocytopenia, a spurious decrease of platelets in vitro due to ethylenediaminetetraacetic acid (EDTA), reported in COVID-19 patients that have led to arterial occlusive events and death [1]. Idiopathic and thrombotic thrombocytopenic events following COVID-19 have been documented, largely in elderly patients (>50 years) with moderate to severe disease [1,6]. One mechanism proposed for the cause of thrombocytopenia in SARS-CoV-2 patients is abnormal hematopoiesis (Figure 1). The viruses in the coronavirus family can infect bone marrow cells. It has been shown that HCoV-229E, a coronavirus, can enter bone marrow cells by binding to CD13 expressed on host cells and resulting in apoptosis and growth inhibition. This apoptosis results in decreased platelet production and eventually thrombocytopenia (Figure 1). It is speculated that the antigen of HCov-229E that binds CD13 has similarities with SARS-CoV-2 antigens. This results in a similar mechanism of thrombocytopenia in patients with COVID-19 [7]. Immune thrombocytopenia (ITP), an acquired hemorrhagic diathesis of impaired production, is due to immune-mediated destruction or increased splenic sequestration of platelets and may be primary (idiopathic) or secondary (infections, HIV infection, medications, connective tissue diseases, malignancies, or secondary to vaccination) [8] (Figure 1). COVID-19 infection is also associated with immune thrombocytopenia, also termed immune thrombocytopenic purpura (ITP), which is slightly more prevalent among males, with elderly males more prone to ITP. ITP mostly occurs 2–3 weeks after the onset of COVID-19 symptoms [6,9]. ITP occurs not only after natural COVID-19 infection but may also occur after COVID-19 vaccination [8,10,11]. 

### 3.2. Coagulation Abnormalities

Disseminated intravascular coagulation (DIC) is a very rare but serious systemic disorder characterized by pro-coagulant, fibrinolytic, and consumption coagulopathy that may result in death [12]. Of note, various studies have reported elevated D-Dimer levels, a major sign of DIC progression, in COVID-19 patients. Increased severity and disease progression led to elevated D-Dimers correlating with disease severity, which is a reliable prognostic marker for in-hospital mortality [13]. Further, elevated D-Dimer levels are a predictor for venous thromboembolism but not a specific predictor for thromboembolic events like pulmonary embolism [14]. However, studies have also documented that elevated D-Dimer levels associated with COVID-19 do not meet the criteria of DIC, as prothrombin time, fibrinogen, and platelet levels were not decreased. This notion is supported by the fact that DIC incidence in COVID-19 non-survivors is about 5%, while it is 0% in survivors [13,15,16]. Rates of thrombosis are also six times higher in COVID-19 patients [1,17]. Altered coagulation markers are poor prognostic indicators of COVID-19. Further markers included a low platelet count, prolonged PT, and a low fibrinogen count [18]. COVID-19 patients in different stages were also marked to be more likely to present with hypercoagulability. Studies detected significant amounts of lupus anticoagulant and antiphospholipid antibodies in COVID-19 patients, strong indicators of hypercoagulability [19]. Complicating factors such as cardiac toxicity, pulmonary embolisms, and deep venous thrombosis were reported as more likely in COVID-19 patients [20]. In the late stages of COVID-19, the lab indices of the patients revealed an increase in von Willebrand factor (vWF) and factor VIIIC, both of which are important classic markers of coagulability. Although these lab indices decreased with constant anti-thrombin treatment, elevated D-dimers persisted as poor prognostic markers [21]. Higher ferritin levels were also present in COVID-19 patients, indicating coagulation and anemic abnormalities [22] (Figure 1).

### 3.3. Red Blood Cells and Hemoglobin

Of the major forms of anemia corresponding with COVID-19, anemia of inflammation has been the more common type. Anemia of inflammation is clinically defined as serum ferritin >100 μg/L with a transferrin saturation <20% [23]. Of the 126 patients diagnosed with COVID-19 with anemia, 56% had anemia of inflammation with elevated ferritin levels [23]. It is important to note that anemia of inflammation occurs more commonly in severe COVID-19 infection as major inflammatory markers, including C-reactive protein (CRP) and ferritin, become elevated [24]. Elevated ferritin, a major iron storage protein, due to inflammation disrupts iron homeostasis, leading to decreased production of hemoglobin, culminating in anemia. Anemic patients have been shown to have significantly higher CRP, which is also positively correlated with ferritin levels [24]. A meta-analysis consisting of 57,563 COVID-19 patients across multiple age groups yielded a mean Hb of 129.7 g/L, a value that can be considered anemic given the guideline (<130 g/L) of anemia in males established by the WHO [25]. Additionally, patients with severe COVID-19 had lower hemoglobin and increased red blood cell distribution width (RDW). However, significance was not established when comparing these levels to those with less severe COVID-19.

Collectively, severe inflammation in severe COVID-19 causes adverse hematological effects, including reduced hemoglobin levels, but significant changes in hemoglobin levels or hematocrit have not been reported in mild to moderate COVID-19 [26,27,28]. A change in other hematological metrics, such as RDW, during COVID-19 has been reported, with an elevated RDW correlating to higher mortality [29]. However, given that patients with reduced hemoglobin levels, age, and other comorbid conditions may have an increased risk of acquiring COVID-19, lower hemoglobin levels should be interpreted cautiously [25].

### 3.4. White Blood Cell

About 20–40% of COVID-19 patients have leukopenia, and 3–24% have leukocytosis. There is a strong association between lymphocytopenia and COVID-19 [1,27]. During the second week of infection, there is a characteristic increase in reactive lymphocytes and antibody-secreting lymphocytes [30]. Eosinophils are reduced in 52.9% of patients [1,31]. It is noteworthy that both lymphocytopenia and neutropenia were positively correlated with disease severity [32]. A study by Li et al. [33] proposed eosinopenia as a reliable predictor for SARS-CoV-2 infection compared to leukocyte counts and lymphopenia. Among other studies, significant morphological changes were observed among granulocytes. For example, Nazarullah et al. [34] found that there was an acquired Pelger-Huët anomaly (APHA) in all COVID-19 cases, with monolobate neutrophils noted to be exclusive to COVID-19 cases. In another study, a peripheral blood smear showed various neutrophils with C-shaped, “fetus-like” nuclei with aberrant nuclear projections, as well as large granular lymphocytes and activated monocytes with prominent cytoplasmic vacuolization [35]. Of further interest is the identification of blue-green cytoplasmic inclusions found in neutrophils and monocytes on blood smears obtained from COVID-19 patients. These findings have been observed in the past within the context of acute liver dysfunction and lactic acidosis and indicate a poor prognosis [36]. In a newer study, blue-green inclusions were found in the cytoplasm of neutrophils and monocytes of COVID-19 patients up to 20 days after initial COVID-19 testing. Additionally, these findings were associated with significantly elevated levels of transaminases, lactic acid, and lactate dehydrogenase [37]. While these are all significant observations within the context of COVID-19, the full clinical implications of these morphological abnormalities must be investigated further.

## 4. Pharmaceutical Treatments

### 4.1. Ritonavir-Boosted Nirmatrelvir (Paxlovid)

Paxlovid was granted access under medical use for COVID-19 and its variants in December 2021 for patients 12 years of age or older who weigh more than 88 lbs and are deemed high-risk [38]. The CDC defines high-risk patients as patients over the age of 50, unvaccinated, or having any certain medical conditions that would compromise their ability to recuperate, such as chronic heart disease, immunodeficiency, or chronic obstructive pulmonary disorder. It has been shown to have an 89% reduction in hospitalizations and deaths among unvaccinated patients. The CDC recommends starting the medication immediately after experiencing any kind of symptoms [39].

Under the COVID-19 guidelines, the National Institute of Health (NIH) recommends providers use Nirmatrelvir 300 mg with Ritonavir 100 mg (Paxlovid) PO twice daily for 5 days as the standard of care for any patients without any significant renal impairment [40]. Paxlovid was shown to have an 89% reduction in hospitalization or death compared to placebo among non-hospitalized patients with confirmed COVID-19 in the EPIC-HR trial [39]. Since Ritonavir inhibits CYP3A4, serious drug-drug interactions with the medications are possible. For instance, Paxlovid has been shown to have moderate interactions with anti-diabetic agents such as Glipizide and Glimepiride; thus, caution is recommended [41]. There is also a significantly increased risk of bleeding between Paxlovid and using anti-coagulants such as Warfarin and Rivaroxaban. It was also found to have a risk of rhabdomyolysis and myopathy with Paxlovid and Atorvastatin. Therefore, a strong recommendation has been made for using Paxlovid with caution of side effects with other medications concomitantly [42].

Clinical studies investigating Paxlovid have shown that the triple regimen is the safest and most efficacious in treating COVID-19. However, it does carry the risk of dysgeusia, diarrhea, hypertension, and myalgia. In a cohort study measuring the safety and effectiveness of Paxlovid, Yan, and team established that there was no significant increase in adverse hematological events such as cancer, leukemia, or heart defects among patients with pre-existing hematological and cardiovascular conditions using Paxlovid [42]. However, teams studying adult and pediatric populations did find an increase in liver enzymes, which was rationalized to be due to Ritonavir acting on hepatic metabolism [43]. In conclusion, Paxlovid is a potential and viable option for preventing COVID-19 deterioration among patients with no current increased risk of adverse hematological events. A recent report from Veteran Health concluded that Paxlovid is associated with a reduced risk of symptoms including blood clots, arrhythmias, fatigue, liver and kidney disease, muscle pain, and shortness of breath, further supporting the safety of Paxlovid in COVID-19 treatment and prevention of long COVID-19 [44].

### 4.2. Bebtelovimab

Currently, the only anti-spike monoclonal antibody available for the treatment of mild to moderate COVID-19 is Bebtelovimab. The drug received emergency use authorization from the US Food and Drug Administration in February 2022, after in vitro data suggested its efficacy against the omicron variant [45]. The mechanism of action of Bebtelovimab is that it neutralizes the CoV-2, B.1.1.7, B.1.351, and B.1.617.2 omicron variants [46,47]. Current NIH guidelines suggest the use of Bebtelovimab only after the use of a 5-day oral course of nirmatrelvir plus ritonavir (Paxlovid), a single IV infusion of sotrovimab, or a 3-day course of IV remdesivir [48]. Studies indicate, however, that Bebtelovimab has comparable efficacy to sotrovimab in mild-to-moderate COVID-19 patients [45,49]. Studies on the use of Bebtelovimab in mostly low-risk adults show an insignificant reduction in viral load at 7 days [48,50]. However, it can be given within 7 days after the onset of symptoms due to its activity against B.1.1.529 (o) and its BA.1 and BA.2 variants. The most common adverse effects of Bebtelovimab are rash, pruritus, and infusion-related reactions. There is also a rare indication of more life-threatening hypersensitivity reactions, including anaphylaxis [48,50]. Large cohort studies of more than 1600 individuals taking Bebtelovimab demonstrate hypertension as the most common comorbidity (42.7%) [49]. Of interest, there has been a report of an 86-year-old male with COVID-19 who experienced bradycardia and cardiac arrest immediately following Bebtelovimab infusion. Therefore, it is imperative to closely monitor cardiac signals while administering Bebtelovimab or other monoclonal antibodies for SARS-CoV-2 [46]. Along with the allergic reactions, difficulty in breathing, shortness of breath, low blood oxygen levels, tachycardia or bradycardia, hypo- or hyper-tension, angioedema, hives, vasovagal reactions (e.g., pre-syncope, syncope), and bleeding at the injection site are common side effects associated with Bebtolovimab [51]. At large, the data at present supports the early administration (within 7 days of symptom onset) of Bebtolovimab, in conjunction with other therapies, in patients with hematologic conditions and mild-moderate COVID-19, as its retained activity against the subvariants BA.1 and BA.2 can minimize further progression of symptoms [5].

### 4.3. Metformin

It has been shown that severe SARS-CoV-2 infection in individuals with comorbidities and preexisting conditions can rapidly progress to acute respiratory distress syndrome (ARDS), multiple organ dysfunction syndrome (MODS), septic shock, and eventual organ failure. Among these comorbidities are diabetes mellitus and obesity [52]. SARS-CoV-2 infects cells by interacting with angiotensin-converting enzyme 2 (ACE2) displayed on host cells. It has been shown that patients with obesity have higher expression of ACE2, especially in adipose tissue, which serves as a large reservoir for the virus. Furthermore, genes regulating lipid metabolism in lung epithelial cells can be upregulated by SARS-CoV-2 in a similar way as obesity and diabetes [53]. Endothelial cells in the vasculature express ACE2, which serves as an entryway for the virus. Endothelial dysfunction (ED) follows virus entry and can result in many hematological events, such as venous thromboembolism, microvasculature lung thrombosis, and disseminated intravascular damage. In diabetes, endothelial cells are also exposed to hyperinsulinemia, increased levels of free fatty acids, and hyperglycemia, which can lead to further ED [52].

Studies have shown that proper management of blood glucose levels in patients with COVID-19 results in less disease severity and a reduction in the rate of late complications [52]. Metformin is an antihyperglycemic agent that is commonly used in patients with type II diabetes. Its mechanism of action works by decreasing hepatic glucose production, decreasing intestinal absorption of glucose, and increasing peripheral uptake of glucose. For this reason, Metformin is significant in the reduction of mortality in patients with diabetes and COVID-19. In addition to the endothelial cell protective mechanisms, it has also been shown that Metformin has further beneficial effects in patients with COVID-19. Metformin can blunt many inflammatory pathways that are associated with ARDS and reduce the severity of this late complication [52].

Some of the most common side effects of Metformin are diarrhea, dyspepsia, poor appetite, vomiting, lactic acidosis, and a metallic taste. However, long-term use of Metformin can significantly increase the risk of vitamin B-12 deficiency, leading to megaloblastic anemia [54]. Furthermore, some cases have reported the incidence of leukocytoclastic vasculitis affecting the skin and leading to ulcers with metformin use in some patient populations [55]. Therefore, caution should be taken when administering Metformin to diabetic patients with hematological malignancies that contract the COVID-19 virus.

### 4.4. Tixagevimab Plus Cilgavimab

Monoclonal antibody (mAb) cocktails are a treatment modality that has been used in the past for viruses such as the Ebola virus and the rabies virus. Within the context of SARS-CoV-2, Tixagevimab plus Cilgavimab (Evusheld) is the only mAb combination therapy that has been approved for use by the Food and Drug Administration (FDA) and the European Medicine Agency (EMA). Of note is its use as a pre-exposure prophylaxis for COVID-19, which has proven to be useful for use among immunocompromised patients [56]. On 8 December 2021, the FDA granted Evusheld emergency use authorization (EUA) to patients with moderate to severely compromised immune systems and/or a report of severe adverse reactions to a COVID-19 vaccine and/or its components. So far, randomized control trials have been conducted, but their scope of results has been limited. For example, the ACTIV-3 trial in the USA showed no differences in sustained recovery at 3 months with the treatment of Evusheld. However, the treatment led to a lower mortality rate of 9% compared to 12% with the placebo [57]. Another concern regarding the Tixagevimab-Cilgavimab cocktail was its effectiveness against the Omicron variants of COVID-19. In another randomized clinical trial (RCT) from Djnirattisai et al. [58], it was shown that Cilgavimab and Tixagevimab alone and together were ineffective against the BA.1 variant. Furthermore, against BA.2/4/5, cilgavimab showed recovered efficacy, while Tixagevimab was still ineffective [59,60]. Despite these concerning results, a mouse study involving anti-Spike mAb regimens, Tixagevimab-Cilgavimab, showed reduced BA.1, BA.1.1, and BA.2 lung infections in human ACE2-transgenic K18 mice when prophylactically administered [61]. However, these results have yet to be studied in a true clinical context. To add to the weakness of the given trials, all the RCTs were studied in individuals who were not vaccinated and minimally immunocompromised. This raises questions about the true efficacy of Tixagevimab-Cilgavimab, as most prescriptions for this cocktail are indicated for individuals who are severely immunocompromised. As far as adverse effects of Tixagevimab-Cilgavimab, only a few hypersensitivity reactions and myalgia have been reported. Evusheld treatment was officially deemed unauthorized for use by the FDA on 26 January 2023, for its allergenic effects and limited ineffectiveness in treating new SARS-CoV-2 strains [62]. Along with allergic and hypersensitivity reactions, myocardial infarction and heart failure were infrequent in clinical trials evaluating Evusheld for pre-exposure prophylaxis, but in later trials, participants had serious cardiac events. This might be due to the presence of risk factors for cardiac disease or a history of cardiovascular disease in participants, and it was not clear whether these were due to Evusheld. Considering these facts, clinicians should consider consulting an allergist-immunologist prior to administering Evusheld, and more clinical trials on a large scale are needed to unravel side effects [63]. This raises questions about the true efficacy of Tixagevimab-Cilgavimab, as most prescriptions for this cocktail are indicated for individuals who are severely immunocompromised.

### 4.5. Convalescent Plasma Therapy

Convalescent plasma therapy (CP) is a well-established methodology for treating viral infections resistant to traditional drugs. In the case of COVID-19, the treatment mechanism is antibody-mediated targeting of the ACE2 receptor among other viral antigens, a common target for antiviral drugs used to treat COVID-19. A notable use of CP includes the Spanish flu, Ebola, and now in the treatment of COVID-19, particularly in high-risk patients [64,65]. CP is administered by using plasma containing antibodies against disease-specific antigens, among other immune and coagulation products obtained from an affected patient, and transferring it to a patient whose immune system has not had enough time to develop a humoral response [66]. The benefits of using CP over traditional antiviral drugs in the treatment of COVID-19 include the presence of polyclonal antibodies, a key advantage when treating a virus capable of rapid mutations, which may render certain antivirals ineffective [67]. A cohort study of 3368 patients admitted to Yale New Haven Health System showed a nearly 50% reduction in in-hospital mortality and fewer days spent on ventilators in patients with moderate to severe COVID-19 [68]. However, this was primarily the case only in patients who received CP early in the presentation of COVID-19 [68].

There is limited information on the adverse hematological effects of CP overall. A meta-analysis conducted by Snow et al. showed no increase in adverse effects when treating COVID-19 with CP [69]. The most common adverse effects reported for the use of CP as a general antiviral therapy were chills, mild elevation of temperature, and complications with the transfusion itself, such as phlebitis or jaundice [70]. Although CP has been an established treatment for over a century, confounding research regarding its efficacy makes it difficult to evaluate its true benefits. It is necessary to further investigate whether the reduction in in-hospital mortality and other outcomes measuring the efficacy of CP treatment is significant, whether it be via large-scale randomized trials or meta-analyses of numerous cohorts.

### 4.6. Dexamethasone plus Remdesivir

Remdesivir is an antiviral medication that first obtained FDA approval for its use in COVID-19 on 22 October 2020 (FDA 2020). Remdesivir was the first antiviral to get FDA authorization against SARS-CoV-2. Remdesivir is a broad-spectrum antiviral that shows activity against many viruses, such as Ebola, the respiratory syncytial virus, and the Coronavirus family. Remdesivir is a nucleotide analog that gets phosphorylated in the host cell in the triphosphate format (RDV-TP). RDV-TP is then used as a substrate for RNA-dependent RNA polymerase (RdRp), which results in delayed chain termination. RDV-TP resembles Adenosine and competes with it for binding to RdRp [71]. Remdesivir showed a decrease in time to recovery for patients that are hospitalized for COVID-19, with evidence of a lower respiratory infection compared to placebo [72].

Dexamethasone is a potent glucocorticoid that has been used extensively with Remdesivir as the standard of care for managing hospitalized patients with COVID-19. The mechanism of action of Dexamethasone is dose-dependent. The genomic mechanism of action occurs at low doses, and the non-genomic mechanism occurs at high doses of the drug [73]. The genomic mechanism involves the stimulation and suppression of many genes in host cells. Dexamethasone can suppress many pro-inflammatory cytokines that have been linked to SARS-CoV-2 [74]. Furthermore, it can stimulate anti-inflammatory cytokines to further reduce inflammation and the destruction of host cells. The non-genomic mechanisms of Dexamethasone can impair receptor signaling and result in a T-lymphocyte-mediated immune response [75].

It has been shown that the majority of the adverse outcomes associated with COVID-19 are due to severe inflammation, injury caused by ARDS, and diffuse alveolar damage [73]. Studies have shown that the use of dexamethasone with remdesivir has led to shorter recovery times and less mortality compared to either medication alone [76,77]. Some of the common side effects of Remdesivir are anemia, increased liver enzymes, and hypotension. However, Remdesivir has been linked to more cardiovascular side effects such as QT prolongation, bradycardia, and atrial fibrillation [78]. Glucocorticoid use in patients with COVID-19 can result in different hematological side effects based on the individual patient. However, a common hematological adverse effect is steroid-induced cutaneous purpura [79].

### 4.7. Molnupiravir

Molnupiravir is a current oral antiviral drug that has been approved for use against COVID-19. Among the other oral antiviral drugs, it was the first one to demonstrate a significant advantage in reducing hospitalization or mortality in mild COVID-19. Molnupiravir was issued an emergency use authorization by the FDA on 23 December 2021 [80]. Molnupiravir acts as a prodrug when orally administered and is converted to the active nucleoside analog, EIDD-1931, by host esterases. In vitro evidence has shown that Molnupiravir is a potent inhibitor of SARS-CoV-2 replication with an EC_50_ in the submicromolar range [81,82]. In the past, EIDD-1931 has been proven to inhibit various viruses, such as the Ebola virus, Norovirus, and Influenza A and B, among many others. Current pharmacokinetic studies show Molnupiravir should be administered twice daily [83]. However, administration with food may decrease the efficacy of the drug through decreased absorption. Common adverse effects of Molnupiravir include headache and diarrhea. However, 93.3% of these adverse effects were considered mild [84]. Within the context of hematological abnormalities, one study exhibited platelet counts below 50,000 per microliter in one subject who was given Molnupiravir [85]. However, this appears to be a relatively negligible number, as this was 1 among 716 participants who received Molnupiravir. Thus, the decreased platelet count was deemed unrelated to the administration of Molnupiravir treatment in short-term therapy (7–10 days) [86], but thrombocytopenia has been reported with long-term therapy in animal models [87]. Molnupiravir treatment is associated with reduced ferritin levels and increased lymphocytes [86], suggesting decreased viral load and inflammation in patients. While clinical studies are ongoing, researchers appear to be hopeful for improved results. However, the long-term use of Molnupiravir has not been studied, so long-term tolerance/adverse effects are not known at this time [82,88]. Table 1 summarizes the efficacy of therapeutic agents that can be used in COVID-19 patients with hematological conditions.

## 5. Antithrombotic Treatment in COVID-19

COVID-19 is associated with an increased risk of thrombotic events, with a predominance of venous thromboembolism. These thrombotic events are associated with increased disease severity, poor clinical outcomes, and a higher risk of death. The relative incidence of vascular events soon after COVID-19 declines more rapidly for arterial thrombosis than venous thrombosis, but the incidence levels remain high up to 49 weeks and even after discharge [93,94,95]. Thus, there is a need for antithrombotic treatment for thromboembolism during COVID-19. The American Society of Hematology (ASH; Table 2) [96,97] and the International Society of Thrombosis and Haemostasis (ISTH; Table 3) [98] have recommended guidelines to treat thromboembolism in patients with COVID-19, depending on the severity of the illness, to reduce the risk of adverse events, including mortality and thromboembolism.

## 6. How to Manage Blood Disorders

In patients with acute myeloid leukemia (AML), the standard 7 + 3 combination regimen of cytarabine for 7 days along with short infusions of anthracycline for 3 days is recommended [99]. High doses of cytarabine can still be administered post-remission [100]. Patients with acute lymphocytic leukemia (ALL) should be treated conscientiously with high-dose steroids only during the post-induction phase, as steroids are associated with viral shedding and mortality. Furthermore, physicians should take major measures to prevent bacterial and fungal infections. Philadelphia-chromosome-positive ALL patients should be given a tyrosine kinase inhibitor [101]. In patients with chronic myeloid leukemia (CML), extreme caution should be taken during the first 3 months of treatment with tyrosine kinase inhibitors as they may induce therapy-related cytopenia [100]. However, tyrosine kinase inhibitors (TKI) plus steroids have been used successfully to manage patients with both Philadelphia chrompsome-positive ALL and CML in Italy [102]. Myeloproliferative neoplasms (MPN) should be treated with a twice-daily, low dose of aspirin, as MPN are prone to thrombosis. In the case of hospitalization, patients should add low-molecular-weight heparin at standard doses [103]. Strict leukocyte count control with hydroxyurea may also decrease the risk of thrombosis. In patients with indolent lymphomas, Rituximab may be considered a single agent [100]. In patients with CLL, Ibrutinib has been proven to be safe and may have the added benefit of preserving pulmonary function in those with severe COVID-19 [104,105]. ABVD (adriamycin, bleomycin sulfate, vinblastine sulfate, and dacarbazine) is the preferred regimen for Hodgkin’s lymphoma [100,106]. Table 4 summarizes the therapeutics of blood disorders in patients with COVID-19.

## 7. Conclusions

The SARS-CoV-2 infection has immensely impacted all corners of the world, particularly those who are immunocompromised. The COVID-19 pandemic has radically altered what protocols are standard for treatment, and careful consideration must be taken when treating individuals with hematological conditions. Thus, there is a need for constant revision of guidelines as more scientific evidence is released. This review is meant to guide clinicians as they navigate the rapidly changing landscape that surrounds COVID-19 treatment and to aid in the provision of excellent care to high-risk patients with hematological conditions.

## Figures and Tables

**Table 1:** Original research can provide evidence of the efficacy of COVID-19 pharmaceuticals in patients with hematological conditions.

Aim of the Study	Sample	Findings
Ritonavir-boosted Nirmatrelvir (Paxlovid)		
To study the use of Paxlovid in immunocompromised patients [70]	114 hospitalized patients with COVID-19 receiving Paxlovid	Paxlovid has therapeutic potential in high-risk populations, especially when administered early after symptom onset.
Safety and efficacy of paxlovid among serious pediatric cases [89]	5 pediatric cases with the underlying disease treated with Paxlovid	Paxlovid is a feasible option for treating children aged 6–14, with underlying disease, infected with COVID-19.
Bebtelovimab		
To quantify the effectiveness of Bebtelovimab compared to sotrovimab [45]	361 solid organ transplant recipients receiving Bebtelovimab or sotrovimab	Bebtelovimab is similar effectiveness as Sotrovimab. Specifically, this data supports the continued use of bebtelovimab in solid organ transplant patients.
Compare outcomes of high risk patients receiving Bebtelovimab for COVID-19 therapy [49]	3607 high risk (older, immunocompromised, multiple comorbid conditions) treated with Bebtelovimab or ritonavir	Bebtelovimab use is supported for the treatment of COVID-19 in high-risk populations including the immunocompromised.
Metformin		
To analyze the potential benefits of Metformin in patients with COVID-19 and type II diabetes [90].	6937 patients hospitalized with covid and treated with metformin to control their blood glucose levels.	Metformin reduces the risk of mortality in patients with COVID-19 and type II diabetes.
Retrospective analysis of human studies to evaluate the protective role of Metformin in patients hospitalized with COVID-19 [91].	6256 patients from 4 different studies hospitalized with COVID-19, were separated into metformin users and non-users.	Metformin significantly reduced the mortality rate of women hospitalized with COVID-19 (not men). Odds ratio of 0.759.
Tixagevimab plus Cilgavimab		
To quantify effectiveness of tixagevimab-cilgavimab mAb cocktail in hospitalized COVID-19 patients [57]	1417 hospitalized COVID-19 patients were infused with tixagevimab-cilgavimab	No improvement in the primary outcome of time to sustained recovery with Tixagevimab-cilgavimab but was safe and mortality was lower.
Convalescent Plasma Therapy		
Metanalysis evaluating the benefits of convalescent plasma therapy in the treatment of COVID-19 [69]	8027 out of 15,587 patients with COVID-19 received convalescent plasma therapy	No clear change in mortality with convalescent plasma therapy. Additionally, no apparent impact on ICU admissions or mechanical ventilation need in patients with mild COVID-19.
To quantify the effectiveness of convalescent plasma therapy in the treatment of COVID-19 [68]	115 out of 3368 patients with moderate to severe COVID-19 treated with Convalescent plasma therapy	Lower 14-day and 30-day mortality and 50% decreased likelihood of in-hospital mortality. Findings only in early plasma recipients.
Metanalysis to explore effectiveness of convalescent plasma therapy for infectious diseases [65]	Utilized 40 out of 3524 initially selected studies	Convalescent plasma therapy was effective in reducing mortality and had limited serious adverse events (SAE) in most patients.
Dexamethasone plus Remdesivir		
To analyze the 30 day mortality and need for mechanical ventilation in patients hospitalized with COVID-19, treated with dexamethasone and remdesivir [77].	2747 patients hospitalized with COVID-19 broken into two groups. One group received only a standard of care (SOC) protocol. The second group received SOC and Dexamethasone + Remdesivir.	30 day mortality was reduced in the group treated with SOC and Dexamethasone + Remdesivir (7.1% reduction). Mechanical ventilation use was also reduced.
To assess the impact of treatment with dexamethasone, remdesivir, or both in COVID-19 patients [92]	89,297 hospital in-patients with COVID-19 receiving dexamethasone, remdesivir or both	Treatment with dexamethasone, remdesivir, or both in patients with COVID-19 was associated with less neurological complication.
Molnupiravir		
To assess the efficacy and safety of treatment with molnupiravir in non-hospitalized COVID-19 patients [85]	1433 non-hospitalized unvaccinated participants with COVID-19 receiving molnupiravir	Early treatment with molnupiravir was associated with a reduced risk of hospitalization and death in COVID-19 patients. However, adverse events were reported in 30.4% of molnupiravir treated patients and 33.0% of placebo patients, with one incidence of low platelet count in each group.
To assess human safety and tolerability of treatment with molnupiravir in healthy volunteers [84]	129 healthy volunteers receiving molnupiravir in various dosages	Treatment with molnupiravir in healthy volunteers was generally well-tolerated and had very minimal to mild side effects.

**Table 2:** The American Society of Hematology guidelines to treat thromboembolism in COVID-19 patients.

Severity of Illness	Recommendation
Critically ill patients who do not have confirmed or suspected venous thromboembolism	Use prophylactic-intensity over intermediate-intensity anticoagulation (low certainty of evidence) Use prophylactic-intensity over therapeutic-intensity anticoagulation (conditional recommendation based on very low certainty in the evidence about effects)
Acutely ill patients who do not have confirmed or suspected venous thromboembolism or another indication for anticoagulation	Use therapeutic-intensity over prophylactic-intensity anticoagulation (conditional recommendation based on very low certainty in the evidence about effects). Use prophylactic-intensity over intermediate-intensity anticoagulation
Patients being discharged from hospital and do not have confirmed or suspected venous thromboembolism	Use outpatient anticoagulant thromboprophylaxis (conditional recommendation based on very low certainty in the evidence about effects).

**Table 3:** The International Society of Thrombosis and Haemostasis guidelines to treat thromboembolism in COVID-19 patients to reduce risk of thromboembolism, end organ failure, or mortality. COR—class of recommendation; DOAC—direct oral anticoagulant; LMWH—low molecular weight heparin; LOE—level of evidence; UFH—unfractionated heparin.

Antithrombotic Therapy for Critically Ill, Hospitalized Patients	
Critically ill patients hospitalized for COVID-19 (COR: 3, no benefit and LOE:B-R)	Intermediate dose LMWH/UFH is not recommended over prophylactic dose LMWH/UFH
Critically ill patients hospitalized for COVID-19 (COR: 3, no benefit and LOE:B-R)	Therapeutic dose LMWH/UFH is not recommended over usual-care or prophylactic dose LMWH/UFHs
In selected critically ill patients hospitalized for COVID-19 (COR: 2b and LOE:B-R)	Adding an antiplatelet agent to prophylactic dose LMWH/UFH is not well established but might be considered to reduce mortality
Antithrombotic therapy for non–critically ill, hospitalized patients	
Non-critically ill patients hospitalized for COVID-19 (COR: 1 and LOE:B-NR)	low (prophylactic) dose LMWH or UFH is recommended in preference to no LMWH or UFH
Selected non-critically ill patients hospitalized for COVID-19 (COR: 1 and LOE:A)	Therapeutic-dose LMWH or UFH is beneficial in preference to low (prophylactic) or intermediate dose LMWH or UFH
Non-critically ill patients hospitalized for COVID-19 (COR: 3, no benefit and LOE:B-R)	Intermediate-dose LMWH or UFH is not recommended in preference to low (prophylactic) dose LMWH or UFH
Non-critically ill patients hospitalized for COVID-19 (COR: 3, Harm and LOE:A)	Add-on treatment with an antiplatelet agent is potentially harmful and should not be used
Non-critically ill patients hospitalized for COVID-19 (COR: 3, no benefit and LOE:B-R)	Therapeutic-dose DOAC is not effective
Antithrombotic therapy for non-hospitalized patients	
Non-hospitalized patients with symptomatic COVID-19 (COR: 3, no benefit and LOE:B-R)	Initiation of antiplatelet therapy is not effective to reduce the risk of hospitalization or thromboembolism
Non-hospitalized patients with symptomatic COVID-19 (COR: 3, no benefit and LOE:B-R)	Initiation of direct oral anticoagulant (DOAC) therapy is not effective to reduce risk of hospitalization or thromboembolism
Non-hospitalized patients with COVID-19 at higher risk of disease progression (COR: 2b and LOE:B-R)	Initiation of oral sulodexide therapy within 3 days of symptom onset may be considered to reduce risk of hospitalization
Antithrombotic therapy for patients discharged from hospital	
Selected patients who have been hospitalized for COVID-19 (COR: 2b and LOE:B-R)	Treatment with prophylactic dose rivaroxaban for approximately 30 days may be considered to reduce risk of VTE

**Table 4:** Management of blood disorders in the context of the SARS-CoV-2 virus.

Disorder	Treatment
AML	7 + 3 cytarabine and anthracycline regimen
ALL	High-dose steroids post-induction, (avoid bacterial and fungal infection)
CML/Ph+ ALL	TKI + steroids (caution during first three months)
MPN	Aspirin twice daily (+LMWH in hospitalized)
Indolent lymphoma	Rituximab
CLL	Ibrutinib
Hodgkins’s lymphoma	ABVD (adriamycin, bleomycin sulfate, vinblastine sulfate, and dacarbazine)

## Data Availability

Not applicable.
